# TNFRSF1A gen and R92Q and P46L variants. Association with other inflammatory diseases

**DOI:** 10.1186/1546-0096-12-S1-P266

**Published:** 2014-09-17

**Authors:** Berta Lopez Montesinos, Maria Isabel Gonzalez Fernandez, Laura Fernandez Silveira, Inmaculada Calvo Penades

**Affiliations:** 1 Pediatric Rheumatology Unit, Hospital La Fe, Valencia, Spain

## Introduction

Tumour Necrosis Factor (TNF) Receptor-Associated Periodic Syndrome (TRAPS) is an autoinflammatory disease with an autosomal dominant inheritance pattern. Structural mutations in the TNFRSF1A gen tend to have a penetrance higher than 90%, except for p.P46L and p.R92Q variants. The intensity of clinical manifestations in patients with these variants has been shown to decrease or even to disappear in the long term follow-up. It has been found a high prevalence of the R92Q mutation in patients with inflammatory diseases known to have a relevant TNF-alfa involvement.

## Objectives

To describe the inflammatory pathologies associated with TNFRSF1A gen variants P46L and R92Q in 15 patients.

## Methods

All the patients with a TNFRSF1A gen variants, discovered in the context of the study of a suspected autoinflamamtory disease, between 2005 and 2014, were included.

## Results

A mutation in TNFRSF1A gen was detected in 18 patients: 3 structural mutations, 13 R92Q variant and 2 P46L variant. Inflammatory disorders found in the group of 15 patients with a TNFRSF1A variant were: 4 vasculitis (Takayasu arteritis, Schölein-Henoch purpura, ANCA positive vasculitis, polyarteritis nodosa), 3 patients fulfilled criteria for systemic juvenile idiopathic arthritis (2 with macrophagic activation syndrome). In 2 patients a familial mediterranean fever mutation was also found and 2 patients showed high fecal caprotectin levels without inflammatory bowel disease symptoms at that moment.

## Conclusion

Although R92Q mutation has been identified in healthy individuals, recent studies suggest that it may increase the susceptibility to other inflammatory conditions as multiple sclerosis, Behçet's disease, the occurrence of extraintestinal manifestations in Crohn disease and vasculitis.

## Disclosure of interest

None declared.

